# A controlled study on gastrointestinal nematodes from two Swedish cattle farms showing field evidence of ivermectin resistance

**DOI:** 10.1186/1756-3305-7-13

**Published:** 2014-01-08

**Authors:** Marlene Areskog, Sofia Sollenberg, Annie Engström, Georg von Samson-Himmelstjerna, Johan Höglund

**Affiliations:** 1 Department of Biomedical Sciences and Veterinary Public Health (BVF), Section for Parasitology, Swedish University of Agricultural Sciences, Uppsala SE-751 89, Sweden; 2 Institute for Parasitology and Tropical Veterinary Medicine, Freie Universität, Berlin, Germany

**Keywords:** Gastrointestinal nematodes, Macrocyclic lactones, *Ostertagia ostertagi*, *Cooperia oncophora*, Anthelmintic resistance, Controlled efficacy test

## Abstract

**Background:**

Anthelmintic resistance (AR) is an increasing problem for the ruminant livestock sector worldwide. However, the extent of the problem is still relatively unknown, especially for parasitic nematodes of cattle. The effect of ivermectin (IVM) (Ivomec inj.®, Merial) was investigated in Swedish isolates of gastrointestinal nematode (GIN) populations showing signs of AR in the field to further characterise the AR status by a range of *in vivo* and *in vitro* methods.

**Methods:**

Three groups, each of 11 calves, were infected with an equal mixture of third stage larvae (L3) of *Cooperia oncophora* and *Ostertagia ostertagi*. Group A was inoculated with an IVM-susceptible laboratory isolate and groups B and C with isolates originating from ‘resistant’ cattle farms. Faecal egg counts (FEC) were monitored from 0 to 45 days post infection (d.p.i.), and L3 were harvested continuously for larval migration inhibition testing (LMIT) and species-specific PCR (ITS2). At 31 d.p.i., one calf from each group was necropsied and adult worms were recovered pre-treatment. At 35 d.p.i., calves from all groups were injected with IVM at the recommended dose (0.2 mg/kg bodyweight). At 45 d.p.i., another two animals from each group were sacrificed and established gastrointestinal worms were collected and counted.

**Results:**

A few animals in all three groups were still excreting eggs (50-150 per g faeces) 10 days post IVM injection. However, there was no significant difference in the FEC reductions in groups A (95%; 95% CI 81-99), B (98%; 92-100) and C (99%; 97-100) between 35 and 44 d.p.i. Furthermore, LMIT showed no significant difference between the three groups. Approximately 100 adult *O. ostertagi* were found in the abomasum of one calf (group B), whereas low to moderate numbers (400-12 200) of *C. oncophora* remained in the small intestine of the calves in all three groups at 45 d.p.i. PCR on L3 harvested from faecal samples up to 10 days post treatment showed a ratio of 100% *C. oncophora* in the calves inoculated with isolates A and B, whereas C also had 8% *O. ostertagi*.

**Conclusions:**

Overall, this experiment showed that the animals were successfully treated according to the Faecal egg count reduction test (FECRT) standard (≥ 95% reduction). However, several adult worms of the dose-limiting species *C. oncophora* demonstrably survived the IVM treatment.

## Background

Gastrointestinal nematode (GIN) infections in livestock are common world-wide and assessments have repeatedly shown that they can cause considerable live weight gain losses during the first grazing season of calves in Sweden [1-3]. It has also recently been demonstrated that there is a negative interaction between exposure to GIN and individual daily milk yields in Swedish dairy herds, even when the overall exposure is relatively low [4]. In temperate regions of the world such as Sweden, the most important GINs include *Cooperia oncophora* and the more pathogenic *Ostertagia ostertagi*, which are usually present as mixed infections in pasture-based cattle production [5].

The use of modern broad spectrum anthelmintics since their introduction in the 1960s has been a convenient and often efficient method to control parasite infections in grazing livestock. However, recent reports have shown that extensive use of anthelmintics has led to a worldwide spread of anthelmintic resistance (AR) in the cattle industry [6,7]. In Europe, AR to macrocyclic lactones (ML), the market-dominating anthelmintic family, has been reported for cattle nematodes in both the UK [8-13] and Belgium [14]. Widespread resistance was also reported in a multinational European survey including German, Belgian and Swedish farms [15]. In a recently performed two-year Swedish faecal egg count reduction test (FECRT) in cattle, the results indicated that the efficacy of topical ML under Swedish field conditions is insufficient, and that *C. oncophora* is the predominant species surviving deworming [16].

Detection of AR is usually based on the FECRT and resistance in ruminant parasitic nematodes is declared when the reduction after ML treatment is ≤95% and with a lower confidence interval (CI) of <90% [17]. AR in trichostrongyloid cattle nematodes detected by FECRT has been reported against all major anthelmintic classes, i.e. against ML and to a lesser extent also the well-investigated benzimidazoles (BZ) [7]. Multiple or cross resistance to both compounds has also been reported in several cases world-wide (for a review, see [7]). In addition to FECRT, a range of *in vitro* bioassays have recently been developed and validated for detection of AR in cattle nematodes, such as the egg hatch test (EHT), the larval development test (LDT), and the larval migration inhibition test (LMIT) [18,19]. Furthermore, there have been attempts to develop molecular-based tests to identify ML resistance, by investigating nematode-specific P-glycoprotein (Pgp) gene expression [20-23] and altered avr-14B gene transcription patterns [24], although no standardised test for routine screening of AR is available as yet.

The survey by Demeler et al. [15] on the efficacy of ML (Ivomec inj.®) included five cattle herds in central Sweden. The evaluation was made among first season grazing (FSG) cattle some weeks after turnout, and was conducted using the FECRT. It showed unsatisfactory efficacy results, with only one farm achieving acceptable reductions in egg output in 2006. In 2007, the reduction was insufficient on all farms where animals were treated with IVM. Isolates from two of these Swedish farms were collected and maintained for further testing. The aim of the present study was to investigate suspected resistance in these *O. ostertagi* and *C. oncophora* isolates after IVM treatment, by performing a controlled efficacy test but also by using a range of available *in vivo* and *in vitro* methods for further characterisation of the AR and species composition status.

## Methods

### Worm material

Three different worm isolates with mixed *O. ostertagi* and *C. oncophora* L3 were used in this study. Isolate A was obtained from Tierärztliche Hochschule, Hannover (TiHo), but was originally identified at the Central Veterinary Laboratory in Weybridge, UK, in 1997. It is a well-investigated laboratory strain that has been repeatedly passaged in calves and with no history of being refractory to treatment with any anthelmintics. Isolates B and C were obtained from two Swedish farms and showed poor reductions to IVM according to FECRT, with 84% (95% CI: 67-95) and 85% (62-99) reductions, respectively, at day 7 [15]. The two Swedish isolates were obtained by collection of faecal samples from FSG at the two farms, mixing them with vermiculite and then incubating them under moist conditions for 2 weeks at 25°C. L3 were harvested by the inverted cover glass technique, and larval cultures were identified according to the morphological key in Borgsteede and Hendriks [25]. After harvest, L3 were passaged once and propagated at SLU’s research department Götala, in naïve dairy cattle that never had access to pasture and were checked for egg shedding prior to experimental infection.

### Housing and animals

The infection trial was performed during seven weeks in February to April 2009 at SLU’s research department Kungsängen, with dairy cattle born and bred on the farm. A total of 33 calves were randomly allocated based on weight and sex to three groups, each with three male calves and eight heifers of the Swedish Red and White breed. All animals were between 3 and 7 months old, weaned but with no experience of grazing, and were checked for GIN egg shedding prior to experimental infection. All calves were weighed prior to the start, then at 21, 35, 51 days post infection (d.p.i.) and when euthanized. Weight varied from 81 to 234 kg, with an average weight at 127 kg at the start of the trial. The calves were allocated to different indoor pens, with 8-9 animals in each. The pens had automatic cleaning systems, with a slowly moving rubber mat removing manure. No changes were made to the diet when the calves were participating in the experiment.

### Experimental infection and anthelmintic treatments

On day 1, each calf was infected with ~40,000 L3 of *O. ostertagi* (50%) and *C. oncophora* (50%) in a small volume of water. Isolates A, B and C were administered to calves in groups A, B and C, respectively. At 35 d.p.i., animals with patent infections were treated with IVM (Ivomec® vet.) as subcutaneous (s.c.) injections. Dosage was according to standard recommendations, 1 mL per 50 kg body weight or 0.2 mg IVM per kg. Animals were treated and the trial was performed according to formal institutional ethical approval (C276/8).

### Sampling and laboratory procedures

Individual faecal samples were collected rectally on 23 occasions between 0 and 45 d.p.i., with more frequent sampling after the third week. A modified McMaster method [26] was used to determine the number of *O. ostertagi* and *C. oncophora* eggs in 3 g of faeces, giving a diagnostic sensitivity of 50 eggs per gram faeces (epg). In addition, the FLOTAC© method [27] with a sensitivity of 2 epg, based on 10 g faeces and 90 mL saturated NaCl, was used at 35 and 44 d.p.i. for comparison. The anthelmintic efficacy of the drug was interpreted through the FECRT, by comparing the results of two different formulae [28]. The first one was based on each group’s arithmetic mean reduction: FECR3 = 100 × (1 − [T2/T1]), using arithmetic means and with no control group [29], where T is treated hosts and the faecal samples were taken pre-treatment or at the moment of treatment (1) and X days post-treatment (2). Individually based FECR formulae were also calculated using before and after treatment individual evaluations in treated hosts: iFECR3 = (1/*n*) ∑ (100 × (1 − [T_*i2*_/T_*i1*_])), where T_*i2*_ is post-treatment and T_*i1*_ is pre-treatment epg in host *i* from a total of *n* hosts. Each host served as its own control. The FECR data were compared (repeated measures MANOVA) using SAS JMP software (version 10.0.2) and the Minitab statistics programme (version 15) and considered significantly different at p < 0.05.

L3 were also cultured by pooling and mixing 10-20 g of each faecal sample with vermiculite and incubating for 2 weeks at 26-27°C. The number of L3 was then counted and identified according to the morphological key in Borgsteede and Hendriks [25].

### Total worm counts

At 31 d.p.i., four days before IVM treatment, one animal per group (A, B and C) was euthanized intravenously with pentobarbital (ex tempore 300 mg/mL, 60 mL/calf). Another two animals per group were euthanized at 45 d.p.i., 10 days post IVM treatment. The abomasum and approximately 7 m of the proximal small intestine (duodenum, jejunum) were separated, truncated, and their contents emptied into individual beakers. The mucosal surfaces were carefully washed with tap water, and the total volume of each calf’s bowel contents was adjusted to 4 L. For each calf, four 20-mL subsamples were then collected under constant stirring from every vessel with bowel or abomasum contents giving a detection level of >200 worms in the original sample [3]. The abomasal mucosa was scraped off into a separate bucket and digested for 6 h at 40°C in a solution with 17 mL HCl (37%) and 10 g pepsin in 1 L H_2_O. The total volume was adjusted to 2 L after digestion and subsampling as above, giving a diagnostic sensitivity of >100 worms. Sub-samples were stored at -20°C, stained with Lugols iodine and examined to count and differentiate worms. Results were compared via one-way ANOVA and boxplots of worm counts (Minitab® 15.1.30.0. software) and considered significantly different at p < 0.05.

### Larval migration inhibition test

Larvae from faecal cultures (21-32 d.p.i.) from each group were obtained via the inverted cover glass technique to ensure that only viable L3 were used. Larval suspension was adjusted to approximately 100 L3 per 20 μL. IVM (MW 871) was purchased from Sigma (I8898). A stock dilution of IVM 10^−2^ M (8.71 mg/mL) was made in dimethyl sulphoxide (DMSO, 100%) and a further dilution into 10 different concentrations of IVM ranging between 10^−5^ and 5 × 10^−10^ M.

The LMIT was carried out with sheathed larvae following the method of Demeler et al. [19]. L3 were incubated in the gradually increasing concentrations of IVM at 28°C in a 24-well plate (incubation plate). After 24 h, the whole contents of each well (liquid + L3) were transferred onto sieves (mesh size 28 μm) suspended on rows 1 and 3 of a 24-well Bacto agar (1.5%) coated plate (migration plate), and allowed to migrate for a further 24 h at 28°C. For every incubation plate, two migration plates were used. After 24 h, migrated L3 in the wells of rows 1 and 3 and non-migrated L3, flushed into corresponding wells of rows 2 and 4, were counted under a stereo microscope. Each isolate was tested over a range of 10 drug concentrations in duplicate (two migration plates per isolate), and negative (water, no drug) and positive (stock solution) controls were run in duplicate on each plate. The percentage of non-migrated L3 in the total amount of L3 was calculated for the controls and every IVM concentration tested. The data were analysed according to Demeler et al. [19] using a logistic regression model to determine LC_50_/LC_99_ with GraphPad Prism® software (version 5.02). The EC_50_ values, the 95% confidence intervals and R^2^ values were calculated, and the three groups compared accordingly. Differences were considered significant at p < 0.05.

### Species-specific single larvae PCR (ITS2)

Genomic DNA from L3 of unknown species (*C. oncophora* or *O. ostertagi*) was isolated from single L3 for traditional PCR. This was carried out via a crude method using proteinase K and 10 mM Tris pH 7.6 in the wells of PCR plates, with minor modifications, according to Zarlenga et al. [30]. In brief, the temperature and incubation time were reduced to 52°C and 1 h, instead of 65°C and 3 h, and 800 μg mL^−1^ proteinase K was used instead of 10 μg mL^−1^. For each *plate*, ~ 50 single individual L3 were tested to determine whether they belonged to *C. oncophora* or *O. ostertagi,* using species-specific primers targeting the ITS-2 ribosomal DNA gene, as described by Schnieder et al. [31] and Höglund et al. [32]. The primer sequences used were *Cooperia* ITS2 Forward 5’ TAA TGG CAT TTG TCT ACA TCT 3’, *Cooperia* ITS2 Reverse 5’ ATG ATA ACG AAT ACT ACT ATC T 3’, *Ostertagia* ITS2 Forward 5’ GTC GAA TGG TAT TTA TTA CT 3’ and *Ostertagia* ITS2 Reverse 5’ TTA GTT TCT TTT CCT CCG CT 3’. The reaction mixtures contained 25 μL volume with 2.5 μL 10 × Buffer (10 mM Tris-HCl pH 8.3, 50 mM KCl, 1.5 mM MgCl_2_ ), 0.5 μL Forward primer (10 pmol), 0.5 μL Reverse primer (10 pmol), 0.5 μL dNTP (0.2 mM), 0.1 μL Polymerase AmpliTaq (0.5 U), 19.9 μL H_2_O, and 1 μL genomic DNA. Samples were run in an Applied Biosystems 2720 Thermal Cycler. Cycling conditions were denaturation at 94°C for 2 min followed by 30 cycles of 94°C for 30 s, 55°C for 30 s and 72°C for 1 minute, followed by 3 min at 72°C. A 6 μL portion of the PCR product was separated on 1% agarose gel (GelRed, Biotium) to *check* the PCR reactions. Bands were documented using UV illumination and digital imaging system (Biorad). PCR products were then purified and amplicons sequenced with BigDye Chemistry (Applied Biosystems) before analysis on an ABI PRISM 3100 Genetic Analyzer (Applied Biosystems).

## Results

### Faecal egg count reduction test

**Figure 1:**
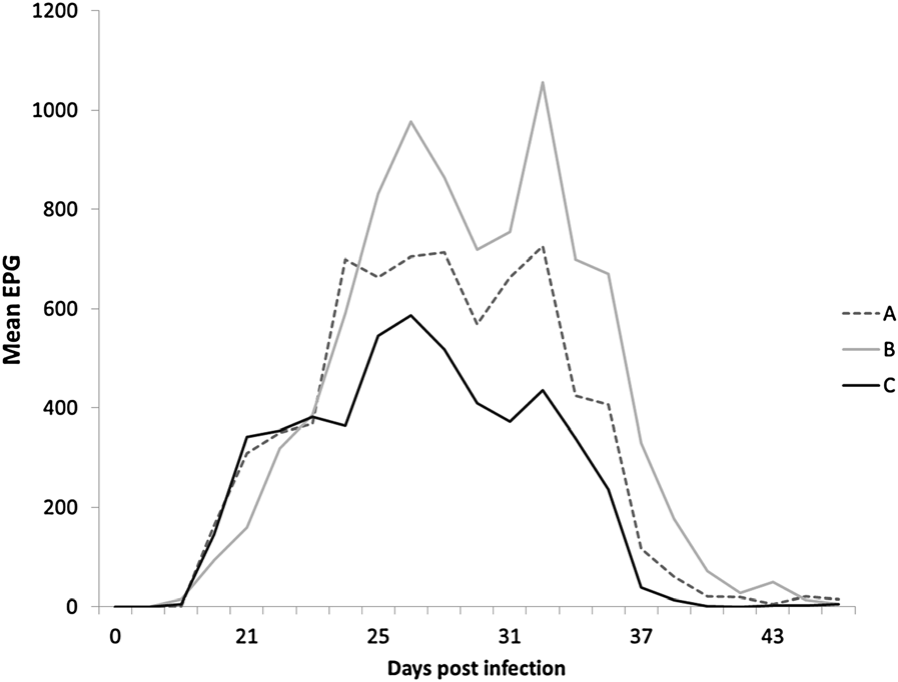
**Faecal egg count patterns.** Mean faecal egg count patterns over time (McMaster method), showing a significant difference (p = 0.002) in egg shedding patterns between isolates (groups) A, B and C from 1-45 d.p.i. However, there was no significant difference (p = 0.34) when comparing the egg shedding levels of the three groups before treatment, 1-35 d.p.i. Group A was infected with a propagated IVM-susceptible laboratory isolate and groups B and C with two different Swedish field isolates showing phenotypic clinical IVM resistance in field trials. All calves were treated with injectable IVM at 35 d.p.i.

**Figure 2:**
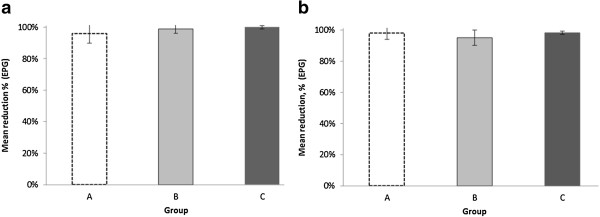
**Mean egg output reduction.** Mean egg output reduction (95%CI) measured by **(a)** the McMaster method and **(b)** the FLOTAC method in isolates 9 days after treatment (44 d.p.i.) with IVM. Calves in group A were infected with a propagated IVM-susceptible laboratory isolate and those in groups B and C with two different field isolates showing phenotypic clinical IVM resistance in field trials. Reductions were similar in all three groups.

The FEC from 0-35 d.p.i. revealed patent infection, with a highest individual egg output of 2350 epg (McMaster) and a highest total mean (McMaster) of 725 (±246), 1055 (±518) and 435 (±219) epg in groups A, B and C, respectively, at 32 d.p.i. All animals except two (100 epg, <50 epg) had a pre-treatment FEC of the recommended limit 150 epg or above at 35 d.p.i. when analysed with the McMaster method [17]. FEC revealed a significant difference (p = 0.03; Log FEC, repeated measures MANOVA) in egg shedding patterns between isolates A, B and C from 1-45 d.p.i. However, there were no significant differences (p = 0.34) when comparing the egg shedding levels of isolates A-C before treatment, i.e. 1-35 d.p.i. (Figure 1). The group-based mean reductions (arithmetic means) between 35 and 44 d.p.i. were: Group A: 95% (95% CI 81-99), group B: 98% (92-100) and group C: 99% (97-100), based on FEC with the McMaster method. Arithmetic means calculated on individual reductions (Figure 2a) showed similar results in groups A (96%), B (99%) and C (100%) with the McMaster method and also in groups A (98%), B (95%) and C (98%) using FLOTAC data (Figure 2b). Statistical analysis (ANOVA) showed no significant (p = 0.33) differences between the reductions in the three groups (McMaster) from 35 to 44 d.p.i. Some animals still excreted eggs (50-150 epg) at 45 d.p.i, 10 days post anthelmintic treatment, in all three groups. Morphological examination of individual L3 from pooled cultures indicated that only *C. oncophora* was present 8-10 days after IVM injection.

### Adult worm recoveries

**Figure 3:**
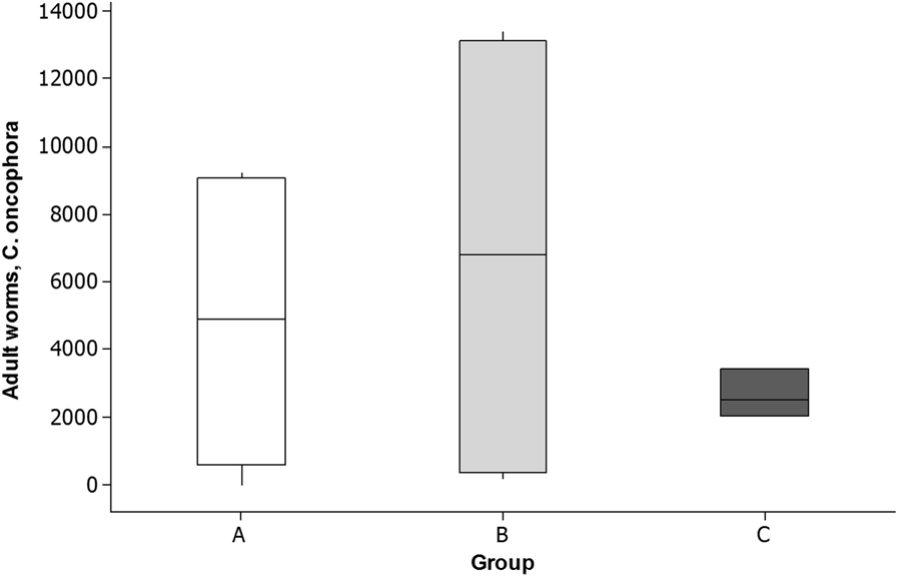
**Adult *C. oncophora *recovered at necropsy post treatment.** Boxplot of estimated numbers of adult *C. oncophora* worms recovered at necropsy of calves 10 days after injection (45 d.p.i.) with IVM. Calves were previously infected with a mixture of *C. oncophora* and *O. ostertagi,* representing isolates with different deworming histories. Isolate A was propagated IVM-susceptible, whereas B and C were two different field isolates showing phenotypic clinical IVM resistance in field trials. Boxplots show larger variation within groups than between groups.

At necropsy 10 days after IVM treatment (45 d.p.i.), ~100 adult *O. ostertagi* were found in the abomasum of one single calf in group B, but between 400 and 12, 200 adults of *C. oncophora* remained in the small intestine of all treated animals (Table 1). The worm burden of groups A-C showed no significant differences (p = 0.74), and box plots (Figure 3) showed larger variation within groups than between groups.

**Table 1:** Calf epg data and recovered worms before and after IVM treatment

**Treatment**	**Calf**	**Origin**	***O. ostertagi***		***C. oncophora***		**epg**
			**Males**	**Females**	**Males**	**Females**	
Before IVM	A1	TiHo	2070	3230	6470	5270	750
	B1	Sweden	570	670	9070	13600	1000
	C1	Sweden	900	2900	1930	3070	400
After IVM	A2	TiHo	0	0	3330	5730	150
	A3	TiHo	0	0	130	400	<50
	B2	Sweden	0	0	6000	6200	50
	B3	Sweden	100	0	200	200	<50
	C2	Sweden	0	0	600	1600	<50
	C3	Sweden	0	0	1000	2000	<50

Calf epg data from day of slaughter, and estimated numbers of adult worms recovered at necropsy from calves before (31 d.p.i.), and 10 days after (45 d.p.i.) s.c. injection with ivermectin (Ivomec®, Merial). Calves were previously each infected with *C. oncophora* and *O. ostertagi,* representing isolates with different deworming history. Calves in group A received equal mixtures of susceptible laboratory maintained *C. oncophora* and *O. ostertagi* from TiHo, whereas calves in groups B and C received cattle nematodes from two different farms in Uppland, Sweden, showing phenotypic clinical IVM resistance in previous field trials.

### Larval migration inhibition test

**Figure 4:**
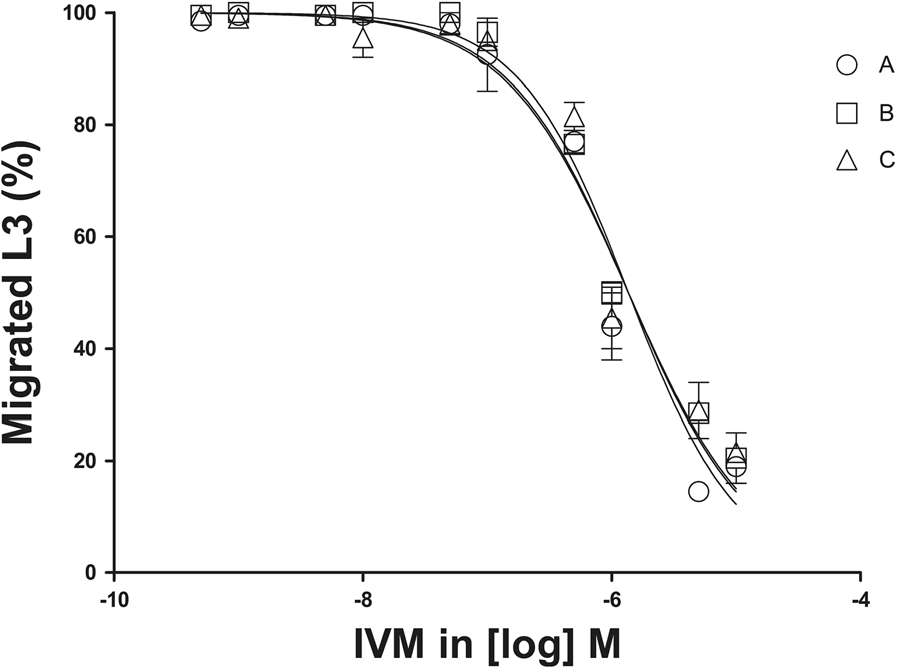
**Dose-response curves, larval migration inhibition test.** Dose-response curves of the data obtained in LMIT with the three different isolates of L3 larvae, showing no significant difference between groups. Calves in group A were infected with equal mixtures of laboratory-maintained *C. oncophora* and *O. ostertagi* from TiHo, and those in groups B and C with cattle nematodes from two different farms in Uppland, Sweden, showing phenotypic clinical IVM resistance in previous field trials.

The EC_50_ values and 95% CI (Table 2) obtained from L3 representing the three different isolates showed no significant differences between groups A-C (p = 0.06). The dose-response curves were also similar for all three groups, as shown in Figure 4.

**Table 2:** Larval migration inhibition test

**IVM**	**A**	**B**	**C**
EC_50_	1.1 μM	1.5 μM	1.5 μM
Conf.-intervals	0.9-1.4 μM	1.3-1.9 μM	1.2-2.1 μM
*R*^*2*^	0.97	0.98	0.95

Results obtained in the LMIT for isolates A, B and C with IVM, including the EC_50_ values, estimated 95% confidence intervals and R^2^ values.

*p* = 0.06.

### Species-specific single larvae PCR (ITS2)

The results from the L3 PCR are shown in Table 3. Group C was the only group with calves still shedding *O. ostertagi* (8%) nematode eggs 7-10 days post IVM treatment (pooled from faecal samples 42-45 d.p.i.). Results from cultured faecal samples collected 4 days before s.c. IVM injections showed about equal proportions of *C. oncophora* and *O. ostertagi* except in group B, where *C. oncophora* was the predominant species (82%) pre-treatment (Table 3).

**Table 3:** Species-specific single larvae PCR (ITS2)

	**Before IVM**	**7-10 days post IVM**
**Group**	**% *O. ostertagi***	**% *O. ostertagi***
A	55	0
B	18	0
C	59	8

Results (% *O. ostertagi* ) from cultured larvae collected separately from groups A, B and C, 4 days before s.c. IVM injection (31 d.p.i.) and 7-10 days post IVM treatment (42-45 d.p.i.).

## Discussion

In this study, we performed a controlled efficacy test with additional available *in vivo* (e.g. FECRT) and *in vitro* methods (LMIT and species identification of surviving larvae by PCR), in order to further investigate previously detected anthelmintic resistance (AR) against Ivomec inj.® (IVM) in two nematode isolates from two Swedish dairy farms. This is the first extended investigation of ML resistance in Sweden under experimental conditions, and the results are somewhat ambiguous.

As in a Belgian survey by El-Abdellati et al. [33], we failed to confirm suspected AR (<95% FECR), regardless of the diagnostic sensitivity of the egg counting method used. Only *C. oncophora* were found in the coprocultures morphologically investigated post treatment, whereas PCR identification of L3 showed that group C calves also shed *O. ostertagi* (8%) post treatment. The adult worm recoveries increased our understanding of the current worm burden, showing considerable quantities of surviving *C. oncophora* but only modest amounts of *O. ostertagi*. Lifschitz et al. [34] have shown that the concentration of IVM is lower in the intestinal mucosa than in the abomasal mucosa, which are the predilection sites of *C. oncophora* and *O. ostertagi*, respectively. The pharmacokinetic properties of IVM may to some extent explain why *C. oncophora* has a higher resilience to the drug than *O. ostertagi*, and why it is the dose-limiting species. Our results from the LMIT showed approximately 10-fold higher EC_50_ values than obtained previously by Demeler et al. [18,19], but in contrast to those studies we tested material with mixed species larvae, which made comparisons difficult.

A deviating finding was that the adult *O. ostertagi* recovered at necropsy came from group B calves, whereas the larvae from shed eggs post treatment came from group C calves. In theory, the lack of shed *O. ostertagi* eggs in group B could be explained by the fact that the nematode uterine muscle is one of the most susceptible target organs for ML [35]. This could mean that the drug may temporarily suppress nematode egg laying, even though adult worms survive treatment. Although we failed to recover adult *O. ostertagi* from the two slaughtered calves in group C at necropsy, L3 in faecal cultures from the same group were observed post treatment according to the species-specific single larva PCR. The most likely explanation is that these fecund females were harboured by another calf in group C that was not slaughtered.

According to the FECRT standard [17,36], all three isolates investigated in this pen trial were successfully treated, with reductions ≥95%. However, the criterion for suspected resistance, i.e. <90 lower limit of the 95% CI, was fulfilled for isolate A (95%, 81-99), which was included as the “susceptible” control isolate, when calculated on group-based means. It was also equal to 90% in isolate A when means were calculated on individual reductions using the McMaster method, and in isolate B when means were calculated on individual reductions using the FLOTAC method.

The reason for the discrepancy between our results and those from the previous field trial remains unknown and needs to be further investigated. In both trials, we used injectable IVM at the same dose rate. The only difference was that in the field trial [15], animal weight was estimated using girth tape, while the animals in the present pen trial were weighed on scales. The remaining differentiating factor is that the calves described by Demeler et al. [15] were dewormed following turn-out. IVM is a very lipophilic substance with an extensive distribution binding to fatty tissues, including sites of parasite location [34,37-39]. Its long persistence after subcutaneous administration to cattle is also based on the deposition of active drug in fatty tissues [40], which could theoretically affect pharmacokinetic patterns under field conditions, since FSG calves in Sweden are well known to suffer from extensive weight loss during the first month after the transition to feeding on pasture [41,42]. In contrast, the animals in this study did not suffer from weight loss or changes in diet, whereas reduced live weight is frequently observed up to four weeks post turn-out in Sweden [43].

It has previously also been shown that factors affecting the pharmacokinetics of IVM clearly affect the efficacy of the drug [44], but the theory of altered pharmacokinetics as a cause of reduced anthelmintic efficacy is still speculative and few investigations have been carried out. Accordingly, further trials under field conditions are needed to test whether the physiological changes FSG undergo during turnout alter the pharamacokinetics of the drug, and how this reduces the efficacy of treatment. Blood samples from field studies where lack of efficacy in ML treatment has been shown [15] would have been a helpful tool in distinguishing between the effect of deviating pharmacokinetics in the host and the effect of AR in the parasitic nematodes, but unfortunately no such samples were available.

## Conclusions

This study showed that calves experimentally infected with two different GIN isolates defined as resistant according to FECRT were successfully treated, although considerable numbers of *C. oncophora* and also small numbers of *O. ostertagi* survived IVM treatment. Genetic AR may be one of several confounding factors leading to anthelmintic failure, but others, such as differences in the pharmacokinetic profile in the field versus compared with in pen trials, cannot yet be excluded.

## Competing interests

The authors declare that they have no competing interests.

## Authors’ contributions

MA performed the experimental work, analysed the data and wrote the manuscript. SS performed sampling and experimental work. AE performed and analysed PCR data. GvSH and JH designed the study and contributed to interpretation and writing of the manuscript. JH also played a great part in the experimental work and obtained the funding. All authors have read and approved the final version of the manuscript.
